# Propolis Modulates Fibronectin Expression in the Matrix of Thermal Injury

**DOI:** 10.1155/2014/748101

**Published:** 2014-03-11

**Authors:** Pawel Olczyk, Katarzyna Komosinska-Vassev, Grzegorz Wisowski, Lukasz Mencner, Jerzy Stojko, Ewa M. Kozma

**Affiliations:** ^1^Department of Community Pharmacy, Medical University of Silesia in Katowice, 41-200 Sosnowiec, Poland; ^2^Department of Clinical Chemistry and Laboratory Diagnostics, Medical University of Silesia in Katowice, 41-200 Sosnowiec, Poland; ^3^Center of Experimental Medicine, Medical University of Silesia in Katowice, 40-752 Katowice, Poland

## Abstract

The aim of the study was to assess the propolis effect on fibronectin metabolism in the course of burn wounds healing process. A model of burn wound healing of pig skin was applied. The amount of the released glycoprotein was assessed by a surface plasmon resonance. The profile of extracted fibronectin components was also assessed by an electrophoresis in polyacrylamide gel, with a subsequent immunodetection by Western Blotting. Propolis burn treatment decreased the release of fibronectin components from healing wounds in relation to damages treated with silver sulfadiazine. The main reason of decreased extraction of fibronectin components from wounds treated with propolis was a substantial decrease of degradation product release of the mentioned glycoprotein, which was observed particularly from the 3rd to 5th day of the repair. Wounds treatment with propolis demonstrated, especially in relation to damages treated with silver sulfadiazine, the decreased release of synthesized fibronectin molecules. The obtained results suggest that propolis modifies fibronectin metabolism in the course of wound healing process. The influence of propolis is reflected in prevention of fibronectin biosynthesis as well as its degradation in the wound area. The above-mentioned metabolic changes may decrease the risk of complications in the repair wounds process.

## 1. Introduction

Fibronectin (FN) is a high molecular weight, multifunctional glycoprotein that determines the structural integrity and various functions of different organs and tissues [1, 2]. Fibronectin is a dimeric molecule [3], built of two similar but not always identical polypeptide chains [2], consisting of three repeating amino acid motifs, named type I, type II, and type III modules [1]. In the course of wound healing, FN modified from inactive soluble molecule into biologically active form [3] participates in every phase of wound healing [4]—hemostasis, inflammation, proliferation, and tissue remodeling [5]. Fibronectin controls bleeding and limits the extent of tissue damage through clot formation [6]. Moreover, the mentioned glycoprotein is important for platelet activity, for example, adhesion, migration, and proliferation [4]. During the second stage of wound repair—inflammation—FN participates in opsonizing ECM debris and activates macrophages for phagocytizing the destroyed tissue residues [1, 4]. The accumulation of fibronectin in wound matrix also impacts on the next phases of the repair process, since the mentioned glycoprotein stimulates theangiogenesis, collagen biosynthesis, granulation tissue formation, and reepithelialisation [1, 4]. In the course of the remodeling phase FN polymerization determines the composition, stabilization, and turnover of the wound extracellular matrix molecules as well as cell-matrix adhesion [4]. However, little is known about the influence of propolis—one of the most promising candidates for burn wound management [7]—on fibronectin expression in the course of thermal damage regeneration. Propolis is a natural resinous material, collected and used by honey bees for the construction of hives, which presents various biological activities such as immunostimulatory, antioxidant, antimicrobial, antiviral, antifungal, antiulcer, anti-inflammatory, and radioprotective as well as properties responsible for augmenting the effects of certain antibiotics [7–12]. Active compounds of Polish propolis used in experiments underlying this paper are flavonoids, aromatic acids, aromatic esters, terpenes, sesquiterpenes, steroids, bioelements, and enzymes [12, 13]. The most important ingredients of Polish propolis are flavonoids, that is, chrysin, tectochrysin, apigenin, pinocembrin, pinostrobin, pinobanksin, galangin, kaempferol, kaempferide, and quercetin as well as phenolic acids such as cinnamic, p-coumaric, ferulic, caffeic acid, and caffeic acid phenylethyl ester (CAPE) [14, 15]. Nowadays, silver sulfadiazine (SSD) is used as an agent of choice in topical burns treatment; however, it exerts serious adverse reactions leading to neutropenia, erythema multiforme, crystalluria, and methaemoglobinemia [9, 16] as well as cytotoxic effect toward fibroblasts and keratinocytes which may retard wound healing process [17]. In addition to injuries treated with silver sulfadiazine, novel methods of wound management such as electrotherapy, laser irradiation, and ultrasound therapy should be paid attention to [18–20]. The first mentioned method—electrotherapy—based on high voltage stimulation, exerts a bactericidal effect, increases cutaneous perfusion, promotes granulation tissue formation, stimulates migration of epidermal cells, restores natural electric potentials, and improves the healing rates [18, 21–23]. Unfortunately, other results suggest that electrotherapy does not influence the acceleration of total therapy of skin damages and the final phase of wound healing [18, 24]. Another method—laser irradiation—is reported to accelerate the healing process, effectively facilitate wound contraction as well as to modulate the inflammation by reducing the levels of proinflammatory cytokines and increase the levels of anti-inflammatory growth factors [25, 26]. On the other hand, there are critical results suggesting that laser irradiation does not enhance the wound healing process [19]. The last listed novel method—ultrasound therapy—prepares the wound bed for healing by reducing bioburden, enhancing angiogenesis, assisting in debridement of necrotic and devitalized tissues, and stimulating cellular activity [27]. However, the results of examination conducted by Dolibog et al. [20] and by Taradaj et al. [28] may suggest that ultrasound therapy does not influence the repair process acceleration. Taking into account the controversies associated with application of SSD and novel methods, such as electrotherapy, laser irradiation, and ultrasound therapy, in wound healing, the introduction of alternative therapeutic agents/methods, such as propolis application, is needed.

Therefore, given that fibronectin is the key component of the interstitial matrices [3], playing structural and functional roles during wound repair process [2, 4], the aim of the present study was to compare the propolis and silver sulfadiazine therapeutic efficiency using the quantitative and qualitative evaluation of the mentioned glycoprotein expression in the matrix of thermal injuries.

## 2. Materials and Methods

### 2.1. Reagents

The following antibodies were used: monoclonal mouse anti-human fibronectin antibody number 42042, purchased from QED Bioscience Inc., San Diego, California, USA, and goat anti-rabbit immunoglobulin G number A5420, conjugated with horseradish peroxidase, obtained from Sigma-Aldrich, Germany. The following reagents were applied: sodium metaperiodate and hydrazide LC-biotin obtained from Thermo Scientific, USA; standard fibronectin from human plasma, DMSO (dimethyl sulfoxide), sodium dodecyl sulfate, Triton X-100, Brilliant blue R250, glycine, Immobilon P membranes, dithiothreitol, Tween 20 (polyoxyethylene sorbitan monolaurate), and TMB (3.3′,5,5′-tetramethylbenzidine), all supplied by Sigma-Aldrich, Germany; Sephadex G-25 obtained from Pharmacia, Sweden; HEPES (4-(2-hydroxyethyl) piperazine-1-ethanesulfonic acid) supplied by Fluka, Germany; BLOT-QuickBlocker purchased from Millipore, USA; TEMED purchased from ICN Biomedicals, USA; streptavidin-coated sensor chip (SAP) obtained from XanTec Bioanalytics, Germany. All of the remaining, applied reagents were supplied by POCh Gliwice, Poland.

### 2.2. Therapeutic Agents

Apitherapeutic ointment containing the ethanolic extract of Polish propolis (prepared according to the method described by Szliszka et al. [14]) was accepted by the National Institute of Hygiene (certificate number: HZ/06107/00; date: November 4, 2000). 1% (0.01 g/mL) silver sulfadiazine (AgSD) cream, obtained from Lek Poland was used.

### 2.3. Tissue Materials

The study protocol was approved by the Ethics Committee of the Medical University of Silesia, Poland (Nr 6/2004). Four domestic four-month-old, pigs were used for the evaluation of wound repair because of many similarities between pig and human skin. Seventy-two contact burn wounds were inflicted on the right and left flanks of the pigs' body, according to the methods of Hoekstra et al. [29] and Brans et al. [30]. Pigs were housed according to the Good Laboratory Practice (GLP) Standards of Polish Veterinary Law. Animals were divided into control (two pigs) and experimental (two pigs) groups. In the control group, wounds were treated with physiologic saline (NaCl) (one animal) or with a propolis vehicle (another animal) twice a day, for 21 days. Wounds treated with NaCl allowed us to observe the healing process occurring without management. Wounds treated with the vehicle alone allowed us, in turn, to assess its possible impact on the propolis therapeutic effect. In the experimental groups, burns were treated with propolis (one animal) or AgSD (another animal), twice a day, for 21 days. Biopsies, in three replications, were taken from healthy skin on day “0” and from the different wound beds on post-burn days 3rd, 5th, 10th, 15th, and 21st. Analgesics given were ketamine hydrochloride and thiopental sodium. Following the thermal damage, tissues were rinsed with an antiseptic solution and treated with apitherapeutic agent, AgSD, apitherapeutic agent vehicle, and physiologic saline. In the case of burns treated with the apitherapeutic agent, AgSD, and apitherapeutic agent vehicle, the wound beds were covered with 55–75 mm layer of used experimental agents. Then, the injuries were protected with a woven cotton material. The thermal injuries left by the biopsy were protected with the collagen dressing.

### 2.4. Extraction of Tissue Fibronectin

Fibronectin was extracted from dehydrated, degreased, and homogenized tissue material with Tris-HCl buffer, pH 7.2, containing 2 M urea, 4% sodium dodecyl sulfate (SDS), and protease inhibitors (0.005 M EDTA, 0.005 M *ε*-amino-n-caproic acid, and 0.001 M PMSF) for 2 h, at 21°C. Then, samples were centrifuged (21000 ×g, 25 min, 21°C), and the tissue pellet was repeatedly submitted to urea/SDS extraction. Fibronectin was precipitated from combined supernatants by an addition of 100% solution of TCA to its final concentration of 10% and incubation for 12 h, at 4°C. The pellets containing fibronectin were separated by centrifugation (21000 ×g, 25 min, 21°C) and repeatedly washed with 80% ethanol to remove TCA [31]. Then, fibronectin samples were stored at −75°C prior to analyses.

### 2.5. Biotinylation of Antibody against Fibronectin

Antibody against fibronectin was dissolved in 0.1 M acetate buffer (pH = 5.5). The obtained solutions were cooled and protected from light and then mixed with an equal volume of 0.02 M sodium metaperiodate in 0.1 M sodium acetate buffer (pH = 5.5). Subsequently, the samples were incubated for 0.5 h at 4°C and then subjected to gel filtration on Sephadex G-25 equilibrated in PBS buffer, pH = 7.2. 1 mL fraction eluting at column void volume and containing anti-fibronectin antibody was collected and mixed with 111 *μ*L of 0.05 M solution of hydrazide LC-biotin in DMSO. The biotinylation of antibodies was being conducted for 2 h, at room temperature. Next, free biotin was removed by dialysis against distilled water and modified anti-fibronectin antibody was lyophilized [32]. The efficiency of antibody biotinylation was controlled with special EZ biotin quantitation kit (Thermo Scientific).

### 2.6. Quantification of Fibronectin in the Hydrolyzates of Burn Wounds

The assessment of fibronectin content in the tissue material derived from healing postburn wounds was made by surface plasmon resonance (SPR) measurement in SPRINGLE instrument (Autolab, the Netherlands) [33]. For this purpose, the biotinylated anti-fibronectin antibodies were immobilized onto streptavidin-coated sensor chip (SAP from XanTec, Germany) and exposed at 21°C to components extracted from tissue with urea/SDS solution. The binding was conducted in 0.01 M HEPES buffer, pH 7.4, containing 0.0034 M EDTA, 0.15 M NaCl, and 0.05% (v/v) Triton X-100. The formation of complexes between the antibody and fibronectin was detected as changes in the SPR signals which were proportional to the amount of bound fibronectin molecules. After binding, the disc surface was regenerated through the dissociation of immune complexes with 0.01 M glycine solution, pH 2.0. The calibration curves were done using various concentrations of standard fibronectin.

### 2.7. The Assessment of Fibronectin in Burn Wounds

Samples of porcine skin extracts were subjected to electrophoresis in a 6% polyacrylamide gel in the presence of SDS according to the method of Laemmli [34]. Prior to electrophoresis, the extract components were treated with 0.04 M dithiothreitol as an agent reducing disulfide bonds. After the electrophoresis, some gels were stained with Brilliant blue R250 and others submitted to electrotransfer on Immobilon P membranes, followed by Western blotting with anti-fibronectin antibodies. Then, the obtained gels and blots were analyzed densitometrically.

### 2.8. Statistical Analysis

Repeated measures analysis of variances (ANOVA) was applied to test the significance of univariate measures of factors with more than two levels (in our research—six levels: day “0” and postburn days—3rd, 5th, 10th, 15th, and 21st) followed by Tukey's post hoc tests, accepting *P* < 0.05 as significant. The special assumption of sphericity (which is a necessary and sufficient condition for the *F*-test to be valid) was verified and held for fibronectin content [35].

## 3. Results

**Figure 1:**
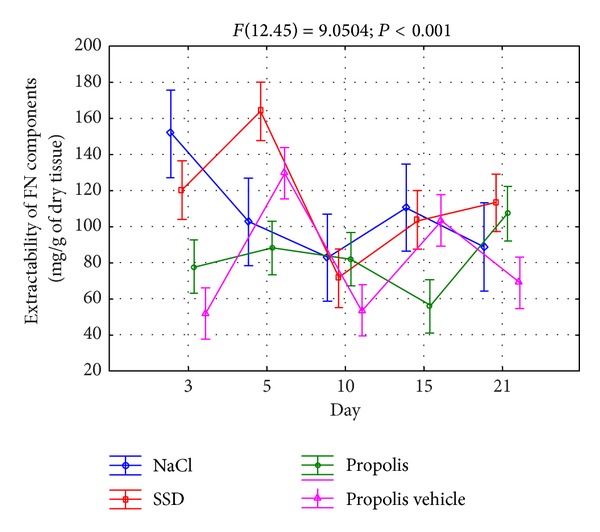
Extractability of FN components isolated from wounds treated with NaCl, SSD, propolis, and propolis vehicle and reacting with reagents against fibronectin.

In order to assess the FN metabolism in the course of differently-treated burn wounds, the extracts of tissue material were collected, which, by using the surface plasmon resonance method, allowed us to assess the content of components reacting with the reagents of this glycoprotein. The obtained data are shown in Figure 1.

As it results from Figure 1, the FN extractability from the place of injury in the course of 21 days of the repair process, regardless of the applied treatment, was significantly diversified demonstrating two maxima. In the case of wounds treated with NaCl, which depict the physiological healing process, the first and particularly abundant release of fibronectin components from the place of damage appeared on the 3rd day of the repair, while the second one, which was significantly lower, appeared on the 15th day (Figure 1). Contrary to damages treated with NaCl, the application of SSD and propolis for wound treatment caused a characteristic delay in the maximal FN extractability with the first apex falling on the 5th day of healing and the second one appearing on the 21st day (Figure 1). However, only in the case of silver sulfadiazine, similarly to the application of NaCl, the first maximum of the fibronectin components' release was significantly higher than the second one (Figure 1), whereas in the case of wounds treated with propolis, the first increase of FN extractability was gentle and considerably lower than the second one (Figure 1). Moreover, the above dynamics of FN release from the burn wounds seems to be caused by a characteristic effect of propolis on the metabolism of this glycoprotein. This conclusion results from a different course of the curves of the mentioned glycoprotein extractability from damages treated with both propolis and the vehicle of apitherapeutic agent (Figure 1).

**Figure 2:**
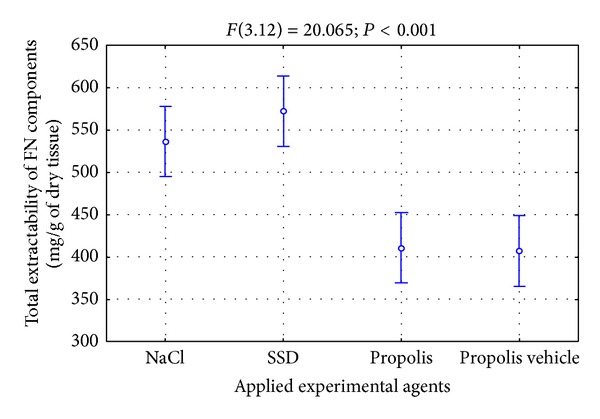
Total extractability of FN components isolated from wounds treated with NaCl, SSD, propolis, and propolis vehicle in the course of healing.

The data concerning the overall number of components reacting with the antibodies for FN, which are released from skin burn wounds during initial 3 weeks of the repair, are shown in Figure 2.

As it results from Figure 2, the FN extractability from the place of damage during initial weeks of healing was not big. Furthermore, characteristically similar amounts of the mentioned glycoprotein were released from wounds treated with NaCl and SSD (Figure 2), whereas wounds treated with propolis and its vehicle were characterized by a lower FN extractability (Figure 2). However, it is worth pointing out that the character of FN metabolism changes in the place of damages is important for the correct course of the healing process. The intensification of proteolysis process of the mentioned glycoprotein in the wound area may lead to releasing biologically active degradation products, for instance, capable of amplifying the inflammatory condition or inhibiting the reconstruction of fibrous weave of the extracellular matrix. The stimulation of FN biosynthesis with a subsequent fibrogenesis of this glycoprotein should intensify the granulation process by stimulating the proliferation, migration, and adhesion of cells and also by restoring the framework of the extracellular matrix.

**Figure 3:**
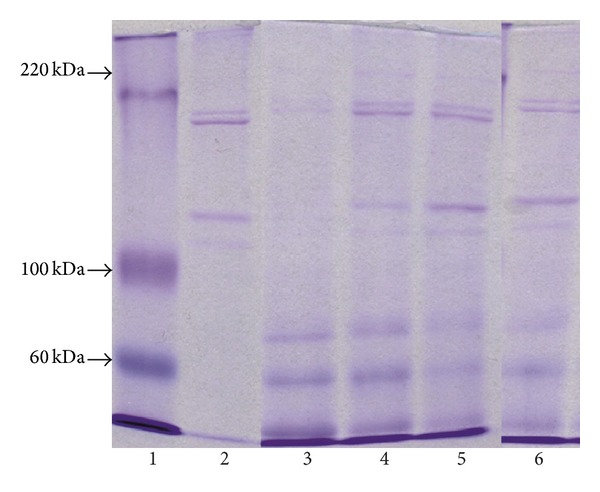
The characteristics of molecular profile of extracts differently treated burn wounds buffered by urea solution and sodium dodecyl sulfate (SDS). Extract components, subjected to dithiothreitol as a factor reducing the disulfide bonds, were subjected to the electrophoresis in a 6% polyacrylamide gel in the presence of SDS. Lane 1, molecular mass markers; lane 2, standard of collagen type I; lanes 3, 4, 5, and 6, components extracted, on the 5th day of healing, from wounds treated by NaCl, SSD, propolis, and propolis vehicle, respectively. The arrows indicate the migration position of standards of known molecular weights (60 kDa, 100 kDa, and 220 kDa).

The character of metabolic processes concerning FN in the area of differently treated skin burn wounds should be displayed in the profile of fibronectin components extracting from the wound beds. The assessment of molecular composition of extracts was conducted using polyacrylamide gel electrophoresis in the presence of SDS and dithiothreitol as a factor reducing the disulfide bonds. The identification of components released from the wounds was done by assessing the immunoreactivity of these molecules with antibodies for FN by Western blotting method. The electropherograms obtained for material extracts taken from differently treated wounds in several different time points of healing of these damages are presented in Figure 3.

**Figure 4:**
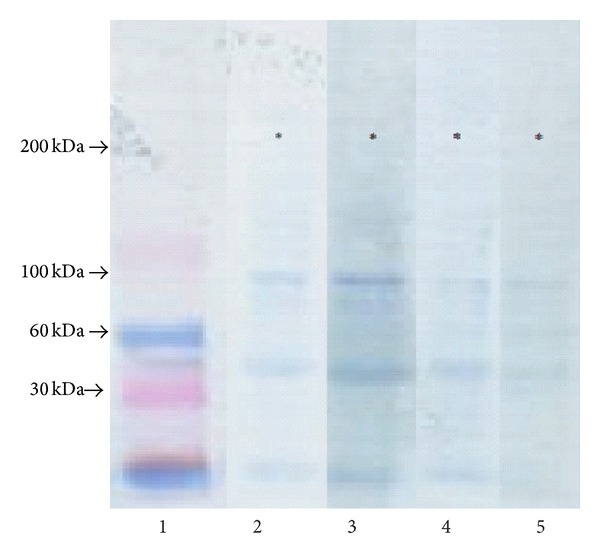
Immunoreactivity assessment of extract components of burn wounds with antibodies against FN. The extract components after electrophoretic separations in gradient polyacrylamide gel 4–15% were subjected to electrotransfer on the membrane Immobilon P and next to the reaction with the antibodies by Western blotting method; lane 1, molecular mass markers, lanes 2, 3, 4, and 5, components released on the 5th day of healing from wounds treated with NaCl, SSD, propolis, and propolis vehicle. The arrows indicate the migration position of molecules of known molecular masses, while the stars indicate the position of fibronectin monomers.

As it can be concluded from Figure 3, all extracts display the similar electrophoretic profiles. Particularly strongly marked components were those which migrated in the front of the separation, as well as bands of three components which are characterized by great electrophoretic mobility and weights equaling about 40, 70, and 100 kDa, respectively, as it can be concluded from the comparison between migration mobility of these components and migrations of reference markers (Figure 3). Despite the above-mentioned molecules, there were also other molecules released from the wound place creating, during electrophoresis, grouped bands in three characteristic doublets (Figure 3). Two of them, created by components with molecular weights of about 110 and 120 kDa as well as of about 190 and 200 kDa, could be clearly seen. Furthermore, the molecules forming the mentioned doublets demonstrated electrophoretic mobilities similar to those characteristic for *α* chains and *β* chains of standard of collagen type I. However, the third doublet was formed by band of components with molecular weights of about 220 and 240 kDa (Figure 3) which were hardly seen on electropherograms. Examination of the influence of extract components with the antibodies against FN by Western immunoblotting method (Figure 4) allowed us to identify the reactivity in the case of elements migrating from the front of resolution components with molecular weights of 40, 70, and 100 kDa and molecules creating the slowest migrating molecular doublet.

The above-mentioned results together with the presented data about molecular weights, characterizing particular components of extracts, suggest that among molecules, released from all tested wounds, there were FN degradation products and native molecules of this glycoprotein which, during electrophoresis in reducing conditions, disintegrated into 2 subunits (monomers). The quantitative assessment of the amount of fibronectin components extracting from differently treated wounds was conducted only on the basis of the densitometric analysis of electropherograms not blots. The reason for doing such an action was significant differences in molecular weights; fibronectin components displayed significant diversity in the velocity of electrotransfer on the membrane which preceded the immunoblotting. Therefore, we suggest that the proportions among fibronectin components on the blots did not reflect the actual quantitative proportions among the molecules in extracts.

**Figure 5:**
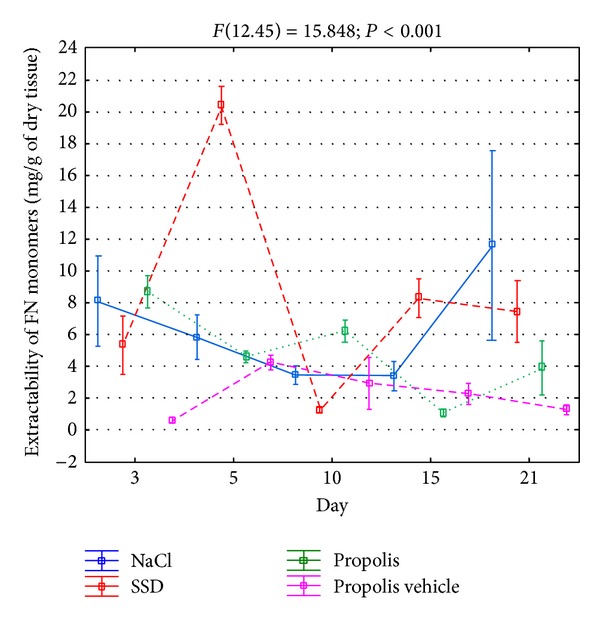
Dynamics of extractability of FN monomers isolated from wounds treated with NaCl, SSD, propolis, and propolis vehicle on the 3rd, 5th, 10th, 15th, and 21st days of healing.

The densitometric analysis of electropherograms confirmed that the native FN molecules constitute only a small percentage of components reacting with antibodies for this glycoprotein in material extracts taken from the area of all tested wounds. Moreover, the presence of native FN molecules in all extracts proves that this glycoprotein is produced in each wound regardless of the way of treating it; only newly secreted molecules from cells are available for extraction which, then, quickly polymerized creating insoluble fibers of the extracellular matrix. As is shown in Figure 5, regardless of the way of treating the wounds, during the healing process of these damages, a diversified release of native fibronectin molecules can be observed.

The release of such components, from wounds rinsed with NaCl, was decreasing between 3rd and 15th days of the repair and then increased twofold on the 21st day. The extractability of native fibronectin molecules from wounds treated with SSD displayed a more strongly marked two-phase character (Figure 5). Particularly intensified release of fibronectin was observed on the 5th day of healing, after which the second, less intense, period of eluting the mentioned glycoprotein came, extending from 15th and 21st days of healing (Figure 5). However, the extractability of fibronectin molecules from wounds treated with propolis had a similar character to that observed in the case of the mentioned glycoprotein on the 21st day of healing. Furthermore, it appears that such dynamics of releasing FN molecule from the wound may be caused by a characteristic influence of propolis on the synthesis of this glycoprotein. This suggestion comes from the fact that there are two different courses of curves of FN molecule extractability from wounds treated with propolis and propolis vehicle (Figure 5).

**Figure 6:**
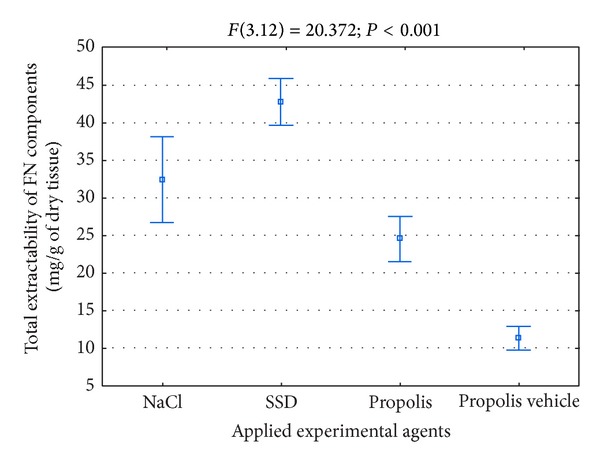
Total extractability of FN monomers isolated from wounds treated with NaCl, SSD, propolis, and propolis vehicle during healing.

The total extractability of native FN molecules from burn wounds in the monitored period of healing was determined as a sum of the mentioned time points. This value reflects the accumulation of fibronectin synthesized in the wound area in the monitored time of healing of tested wounds. As it can be concluded from the data presented in Figure 6, from the wounds treated with propolis, a significantly smaller amount of fibronectin molecules was released in relation to wounds treated with both NaCl and SSD.

**Figure 7:**
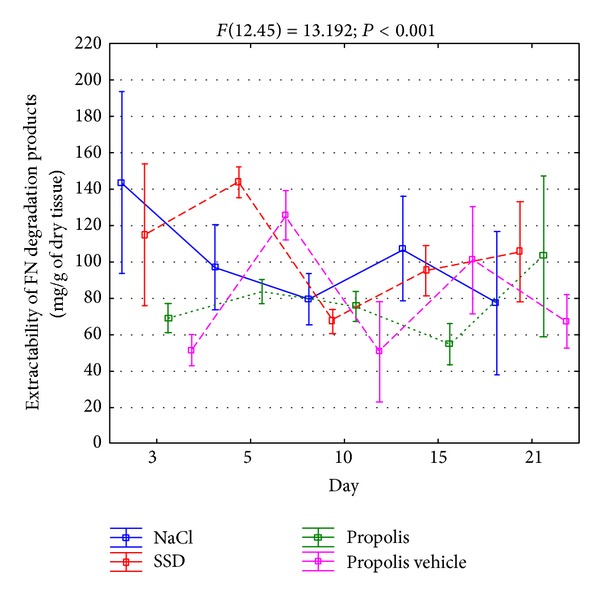
Dynamics of extractability of FN macromolecular degradation products isolated from wounds treated with NaCl, SSD, propolis, and propolis vehicle on the 3rd, 5th, 10th, 15th, and 21st days of healing.

The enzymatic FN degradation in the extracellular space leads to releasing a few, well characterized types of degradation products, among which those whose molecular weight equals 40, 70, and 100 kDa demonstrate a biologic activity of an antagonistic character towards native molecules of this glycoprotein. The extractability of assessed macromolecular products of FN degradation, from differently treated burn wounds in the initial period of healing, is presented in Figure 7.

As can be concluded from Figure 7, irrespective of the way of treating the wounds, the release of FN degradation products during the process of healing was diversified, which suggests the intensity changes of catabolic processes of the glycoprotein in question. The increased release of FN macromolecular degradation products from wounds treated with NaCl and silver sulfadiazine had a clear, two-phase character (Figure 7). First particularly high increase of extractability of the components in question appeared rather early in the course of the repair process which fell on the 3rd day of healing in damages rinsed with NaCl and on the 5th day of the repair in wounds treated with SSD (Figure 7). The second, smaller increase of released products of fibronectin degradation was observed on the 15th day of the repair of damages treated with NaCl and on the 21st day of healing of wounds treated with SSD (Figure 7). However, the curve of extractability of FN degradation products of wounds treated with propolis had a different character. Particularly characteristic was a slight release of these components in the first phase of healing (days 3 and 5), two times lower than that observed in the case of wounds treated with the other agents (Figure 7). A quite significant increase of FN macromolecular degradation products from wounds treated with propolis was seen not until the 21st day of the repair process reaching the values similar to those observed in wounds treated with silver sulfadiazine at the same time of the healing period (Figure 7). It should be also emphasized that the above-presented dynamics of the release of FN degradation products from wounds treated with propolis was most probably a characteristic effect of the apitherapeutic agentactivity on the catabolism of the mentioned glycoprotein. The presented suggestion results from a different course of extractability curves of FN degradation products from wounds treated with propolis and its vehicle (Figure 7).

**Figure 8:**
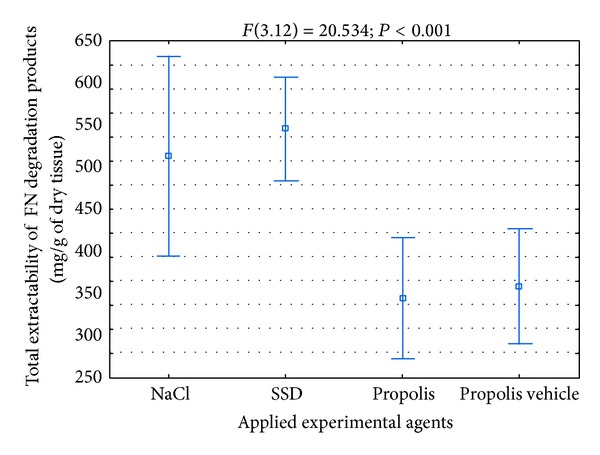
Total extractability of FN macromolecular degradation products isolated from wounds treated with NaCl, SSD, propolis, and propolis vehicle in the process of healing.

The total amount of FN macromolecular degradation products, released from wounds in the course of healing, is very important for the correct course of the repair process. The mentioned amount proves the intensity of FN degradation in the course of the repair process. On the other hand, the presence of the FN degradation products in the healing tissues, taking into consideration the biological action of the latter ones, may upset the course of the repair process. As the presented data in Figure 8 suggest, in the course of healing of wounds treated with propolis, a significantly smaller amount of FN macromolecular degradation products was released than in the case of damages treated with SSD or NaCl.

## 4. Discussion

Wound healing is a complex process which comprises 4 partially overlapping phases: hemostasis, inflammatory phase, proliferation phase, and remodeling. All mentioned phases are indispensable for the repair process; the second and third phase seem to be particularly important for the modification of the process in question [36]. The inflammatory condition, which is already induced during hemostasis, develops during the first day after the injury and remains especially intense until the third day in the case of the correctly proceeding repair process [37, 38].

Around the third day after the injury, the transition of the inflammatory phase to the process of rebuilding the damaged tissue takes place [39]. The transition is caused by the influence of numerous growth factors, secreted by macrophages, which activate the adjacent cells stimulating their proliferation and directed migration. Furthermore, the growth factors, such as PDGF or TGF*β*, stimulate the fibroblasts entering the wound place in order to produce and secrete the extracellular matrix components including fibronectin and different types of collagen. Restoring the extracellular matrix of a specified composition in the wound area is fundamental for the migration of cells as well as for their adhesion and differentiation [38, 39]. All mentioned phenomena, taking place during the proliferation phase of the healing process, lead to formation of a new tissue in the wound area—the granulation tissue. According to the current results, it has been assumed that, in the correctly proceeding healing process, a significantly intense proliferation of cells in the wound place takes place between the third, fourth, and tenth days after the injury, while the fully formed granulation tissue appears in about 15th–20th days of the repair [37]. Thus, applied in the present study time of monitoring the burn wounds, lasting from 3rd to 21st day after the injury, should include the end of the inflammatory phase and the whole proliferation phase of the healing process until the granulation tissue appears. The course of the mentioned phases determines the effectiveness of the repair process. The inflammatory condition is particularly important for the course of healing. On the other hand, the chronic character of the inflammatory condition in the injury place leads to a significant inhibition of the healing process or even to stopping it, which is clinically manifested by incorrectly healing wounds or nonhealing wounds [38–40].

In order to avoid the mentioned complications in the healing process, the damages are subjected to various medical procedures. The classic way of treating such wounds is based on applying silver sulfadiazine [41–43]. However, this compound also demonstrates a series of undesirable local actions such as cytotoxicity against fibroblasts and keratinocytes [44]. The systemic disorders following the silver sulfadiazine application have also been described [45–47]. Beside the application of SSD in the course of injury management, other novel, promising methods, such as electrotherapy, laser irradiation, and ultrasound therapy, of possible application in wound treatment exist. However, SSD application and implementation of novel techniques are not free from certain disadvantages [18–20]. This is the reason why the alternative agents for burn wound treatment are being sought for. One of the examined agents is propolis, a complex, resin-like substance, accumulated and processed by bees. Propolis demonstrates a series of properties which may be favorable for the course of wound healing. Among these properties there are the antibacterial, antivirus, and antifungal activity [48–51]. Moreover, propolis significantly decreases the activity of free radicals in the healing wound beds which favors the repair process itself. This phenomenon was confirmed in our previous studies [51–53]. It was proven that in the tissue samples, taken from the burn wound areas, treated with propolis and silver sulfadiazine, respectively, the expression of free radicals was significantly lower in the case of the first medical substance. Propolis also had a positive effect on collagen metabolism in the area of burn wounds during the healing process, increasing the tissue content of collagen of both type I and type III, which is known to lead to restoring the extracellular matrix and stimulating in this way the granulation tissue [50]. The results obtained in the present study for the first time reveal that the mentioned bee product also modifies the metabolism of fibronectin, which creates the fibrous weave of the extracellular matrix. The influence could be seen in significant differences concerning the extractability of components reacting with the antibodies for fibronectin from the wounds treated with propolis to damages treated with silver sulfadiazine or the wounds treated with NaCl which represent the control and the physiological course of the healing process. The differences in releasing the fibronectin components from the wound area were particularly visible in the initial period of healing (third and fifth days of the repair, resp., related to the control wounds and to those treated with silver sulfadiazine) and also on the fifteenth day after the injury. In the mentioned time points, the release of fibronectin from wounds treated with propolis was significantly lower than in damages treated with the other agents. An important reason of the decrease of extractability of fibronectin components from wounds treated with propolis to the other damages was a significant (about one a half time) reduction in releasing the macromolecule degradation products of fibronectin. Particularly clear differences in releasing such components among wounds treated with propolis and control wounds fell on the third and fifteenth days of healing, while among those treated with propolis and SSD on fifth and fifteenth days of the repair. The observed decrease of extractability of fibronectin degradation products for the healing wounds, treated with propolis, suggests that this bee product may inhibit the disintegration of the fibronectin in the course of the repair process. Fibronectin is catabolized by metalloproteinases (MMPs), particularly by MMP-3 (stromelysin) [38, 50]. These enzymes with zinc ions in the active centre, during the repair of tissue damages, are secreted at first by macrophages and later, under the influence of FGF and PDGF, by cells migrating into the wounds, particularly by fibroblasts which are responsible for the reconstruction of the extracellular matrix in the wound area [37, 40]. Thus, it can be concluded that a particularly high activity of MMPs in the wound area should fall on the third-fifth and fifteenth to twenty-first day of healing. The mentioned dynamics of MMPs activity corresponds with the location of maxima of the extractability of fibronectin macromolecular degradation products from control wounds and those treated with silver sulfadiazine. By contrast, the lack of increased release of the degradation products of the glycoprotein in question, which falls on the third-fifth day of healing in wounds treated with propolis ointment, suggests that this therapeutic agent may regulate the expression and/or activity of MMPs produced by macrophages. It seems that the mechanism of the observed propolis effect may lie in stimulating the production of TGF*β* [36, 37], which is a well-known inhibitory factor of the MMPs expression, as well as in stimulating the production of tissue inhibitors of these enzymes [36, 37, 40].

Our results point out that during healing of propolis treated wounds, especially when related to those treated with SSD, the fibronectin content in wound bed is being decreased. This phenomenon was reflected in the differences of the amount of fibronectin monomers extracted from both types of wounds. These differences were particularly strongly manifested on the fifth, fifteenth, and twenty-first days of the repair. However, in the case of the control wounds and those treated with propolis, the differences in the accumulation of synthesized fibronectin in the wound bed concerned the twenty-first day of healing. Taking into account that propolis significantly decreased the fibronectin degradation during the healing process, the observed changes suggest that this apitherapeutic agent has an inhibiting effect also on the fibronectin biosynthesis. The source of this glycoprotein, synthesized in the damage area mainly during the proliferation phase, is the endothelial cells, mainly fibroblasts [38, 54, 55]. It has been proven that the latter cells, originating from the keloid samples, inhibit the fibronectin biosynthesis in the presence of quercetin, being one of the propolis components [56]. It was also shown that another propolis component, that is, resveratrol, possesses the ability to inhibit the TGF*β*-dependent production of fibronectin in C2C12 myoblasts [57]. The mechanism responsible for the mentioned effect is connected with the activating action of resveratrol on the NAD^+^-dependent SIRT1 histone-protein deacetylase [57]. It is not known, however, if a similar phenomenon is fundamental for the observed propolis effect on fibronectin metabolism in cells (most probably fibroblasts) which are present in the area of healing skin wounds. In fibroblasts, in the conditions of tissue damage repair, the regulation of fibronectin expression takes place not only by the path induced by TGF*β* but also by that stimulated by a group of Wnt ligands which involves *β*-catenin as an intracellular signal transmitter [38]. The presented results pointed out the propolis influence on the fibronectin metabolism in the course of wound healing process poses a question about possible consequences of such an action on the repair itself. In the process of healing, fibronectin fulfills many key functions regulating the cell behavior and being responsible for the formation of the fibrous weave of the extracellular matrix. The three-dimensional fibronectin matrix is the necessary environment for cell migration, proliferation, and differentiation as well as adhesion and apoptosis [54]. The influence of cell surface integrin receptors with fibronectin molecules launching a complex intracellular signalization and determining the expression of proper genes [54] is fundamental for the mentioned activities of cells. The extremely important role of fibronectin in the repair process is visible in the conditions of the glycoprotein intensified degradation, leading to its lack in the cell microenvironment, followed by the disorder in forming the granulation tissue, which, in turn, causes the complications in the healing process or even its total inhibition [54]. It was found that there are close, however not well-known, relations between the fibronectin content in the cell environment and their activity. Hamill at al. [58] found that the decreased fibronectin content in the extracellular matrix stimulates the mobility of skin cells. Similarly, Inoue at al. [59] showed that migrating epithelial cells are characterized by a lower fibronectin expression than those in the resting state. Therefore, the lower content of the glycoprotein in the propolis treated wounds compared to those treated with SSD suggests that the application of the apitherapeutic agent for burn wound treatment may be more beneficial as far as the pace of forming the granulation tissue is concerned. Moreover, this phenomenon may be also supported by the inhibitory effect of propolis on the fibronectin degradation. In fact, it is known that some macromolecular products of fibronectin degradation not only interfere in the cell migration or adhesion to native fibronectin molecules but also have chemotactic properties for inflammatory cells, making the inflammatory phase longer [54, 60, 61].

Despite its effect on the cells, fibronectin also influences the composition and structure of the extracellular matrix. It has been proven that the accumulation of the mentioned glycoprotein in the extracellular space regulates the secretion of other components of this matrix such as collagen type I and type III, tenascin, laminin, and fibrillin [54]. The last mentioned glycoprotein forms fibrillar components of elastin fibers. Furthermore, fibronectin is one of the main factors determining the collagen fibrogenesis [54]. The excessive production and secretion of collagen in fibromatous tissues are preceded by the increased fibronectin accumulation in the extracellular matrix [38, 54]. Thus, the reduced* de novo* fibronectin synthesis in propolis treated burn wounds related to control ones (NaCl treated), which was observed on the twenty-first day of the repair process, may be the factor decreasing the risk of keloid development, being a frequent reason for complications in the burn wound healing [38]. The obtained results indicate that the propolis influence on fibronectin metabolism may be one of the mechanisms because of which this apitherapeutic agent exerts beneficial effect on wound healing process.
